# PREVALENCE AND SOCIOECONOMIC DETERMINATES OF FOOD INSECURITY IN VETERANS: FINDINGS FROM NHANES

**DOI:** 10.1093/geroni/igad104.2465

**Published:** 2023-12-21

**Authors:** Ronna Robbins, Odessa Addison, Elizabeth Parker, Sarah Wherry, Monica Serra

**Affiliations:** South Texas Veterans Health Care System, Flower Mound, Texas, United States; University of Maryland School of Medicine, Baltimore, Maryland, United States; UMSOM, Baltimore, Maryland, United States; VA Eastern Colorado Health Care System, Aurora, Colorado, United States; UT Health San Antonio, San Antonio, Texas, United States

## Abstract

**Objective:**

Determine predictors of the association between being a Veteran and adult food security, as well as to examine the relation of potential covariates to this relationship.

**Methods:**

Data collected during 2011-2012, 2013-2014, and 2015-2016 National Health and Nutrition Examination Survey (NHANES) were pooled for analyses. 1,227 self-reported Veterans (mean age: 57 years) were matched to 2,432 non-Veterans (mean age: 53 years) on age, race/ethnicity, sex, and education. Adjusted logistic regression was used to determine the odds of Veterans having high food security vs. the combination of marginal, low, and very low food security compared to non-Veterans.

**Results:**

Veteran-status had no effect on the proportion of food insecurities between Veterans and non-Veterans reporting high (Veterans vs. non-Veteran: 79% vs. 80%), marginal (9% vs. 8%), low (5% vs 6%), and very low (8% vs. 6%) food security (p=0.11). However, after controlling for covariates (age, race, education, diabetes, cardiovascular disease, hypertension, and depression), Veterans tended to be less likely to have high food security (OR: 0.82 (95% CI: 0.66, 1.02), p=0.07). Further, non-Hispanic Veterans (OR: 0.72 (95% CI: 0.55, 0.95), p=0.02) and Veterans completing some college (OR: 0.71 (95% CI: 0.50, 0.99), p< 0.05), were significantly less likely to experience high food security compared to non-Veterans.

**Conclusion:**

This study supports previous research findings that after controlling for covariates, Veterans tend to be less likely to have high food security. It also highlights ethnicity and level of education as important socioeconomic determinates of food security status in Veterans.

